# Retrospective cohort study: stereotactic aspiration of necrotic tissue versus conservative medical management for malignant MCA infarction

**DOI:** 10.3389/fsurg.2026.1627837

**Published:** 2026-02-10

**Authors:** Xi Zhang, Yao Zhang, Quan Chen, Hang Wu, Yijun Deng, Aidong Zheng, Hongtian Zhang, Maogang Chen

**Affiliations:** 1 The Center of Stroke, Affiliated Hospital 6 of Nantong University, Medical School of Nantong Univeisity, Nantong, JiangSu, China; 2 Department of Neurology, Pukou Hospital of Chinese Medicine Affiliated to China Pharmaceutical University, Nanjing, JiangSu, China; 3 Department of Neurology, Huai’an First People’s Hospital, Huai’an, JiangSu, China; 4 Department of Neurology, Affiliated Hospital 6 of Nantong University, Yancheng Third People’s Hospital, Yancheng, JiangSu, China; 5 Department of Emergency, Yancheng Clinical College of Xuzhou Medical University, Yancheng No.1 People’s Hospital, Yancheng, JiangSu, China; 6 Department of Emergency, Jianhu People’s Hospital, Yancheng, JiangSu, China; 7 Department of Neurosurgery, Chinese PLA General Hospital, Chinese PLA Medical School, Beijing, China

**Keywords:** decompressive hemicraniectomy, large hemispheric infarction, malignant cerebral middle artery infarction, stereotactic aspiration, the elderly

## Abstract

**Objective:**

This study aimed to investigate the efficacy and safety of stereotactic aspiration of necrotic brain tissue in patients aged 61 years and older with malignant middle cerebral artery infarction (MMI).

**Methods:**

A total of 121 MMI patients aged 61 years and older were enrolled retrospectively in this cohort. They were subjected to conservative medical treatment alone or in addition to stereotactic aspiration of necrotic brain tissue between 24 and 72 h after symptom onset. Perioperative outcomes and 6-month follow-up outcomes were observed and evaluated.

**Results:**

Baseline characteristics were well balanced between the conservative treatment group and the aspiration group, except for a higher prevalence of hypertension in the conservative group. The incidence of early cerebral herniation (8.5% vs. 47.3%, *χ*^2^ = 179.797, *P* < 0.001) and death (19.1% vs. 64.9%, *χ*^2^ = 24.110, *P* < 0.001) in the aspiration group was significantly lower than that in the conservative group, and there was no significant difference in the incidence of cerebral hemorrhage and intracranial infection between the groups (*P* > 0.05). At 6-month follow-up, compared with the conservative treatment group, the aspiration group had a higher proportion of patients achieving favorable outcome (mRS 0–3) (25.5% vs. 2.7%, *χ*^2^ = 14.641, *P* < 0.001) and survival without severe disability (mRS 0–4) (55.3% vs. 17.6%, *χ*^2^ = 18.755, *P* < 0.001). Univariable analysis of factors affecting unfavorable outcome (mRS 4–6) showed that the proportion of patients treated with medical therapy alone was significantly higher in the unfavorable outcome group compared to those treated with aspiration(67.3% vs. 32.7%, *χ*^2^ = 14.641, *P* < 0.001). Multivariate binary logistic regression analysis, used to adjust for confounding factors such as atrial fibrillation, diabetes, smoking, GCS and NIHSS scores at 24 h after onset, revealed that treatment with aspiration was an independent factor associated with a 6-month favorable outcome in the elderly patients with MMI (*OR* 51.713, *95% CI* 5.029–531.748, *P* < 0.001). As a sensitivity analysis, ordinal logistic regression showed that the proportional odds ratio (POR) for the conservative group vs. the aspiration group was 6.737 (95% CI: 3.181–14.271, *P* < 0.001) in the univariable model and 11.290 (95% CI: 4.827–26.409, *P* < 0.001) in the multivariable model, confirming that conservative treatment was significantly associated with worse mRS outcomes.

**Conclusion:**

The study showed stereotactic aspiration of necrotic brain tissue could be effective and safe for the elderly patients with MMI.

## 1 Introduction

Malignant middle cerebral artery infarction (MMI) is a common life-threatening neurological emergency. It has a poor prognosis, with a mortality rate as high as 80% even under the optimal medical conservative treatment (1, 2). In the MMI syndrome, rapidly progressive space-occupying brain edema secondary to massive cerebral infarction causes refractory intracranial hypertension, accompanied by rapidly progressive neurological deterioration and the clinical signs of cerebral herniation within 24–96 h after onset (3, 4).

Approximately 50% of patients with MMI are over 60 years old, but no optimal treatment is currently available for elderly patients (5, 6). Decompressive hemicraniectomy (DHC) is currently recognized as a standard therapeutic intervention for MMI. However, for patients aged over 60 years, its effectiveness is limited by severe surgical trauma, stress and the high incidence of complications such as cerebral hemorrhage, intracranial infection, hydrocephalus and sinking skin flap syndrome, etc (7). Moreover, elderly patients often suffer from a decreased cardiopulmonary function, which in turn restricts their tolerance for the surgical procedure. The DESTINY Ⅱ study (5) revealed an unacceptably high mortality rate of 33% at 6 months and 43% at 12 months post-stroke in elderly patients with MMI who underwent DHC. Additionally, the study showed no statistically significant difference between DHC and medical therapy alone in the primary endpoint, which was defined as the ratio of favorable outcome (mRS ≤ 3). Furthermore, survivors of DHC usually undergo a second surgical procedure to close the skull defect (cranioplasty). The procedure not only increases additional medical costs, but also carries a high risk of complications. In the latest guidelines (4, 8), the recommendation level of DHC for treating the elderly patients is low. Considering the limited efficacy, unfavorable prognosis, and high medical expense of DHC, less than 15% of MMI patients who meet the eligibility criteria actually undergo this procedure (9, 10), and this proportion is even lower in elderly patients. Therefore, the development of new safe and effective therapeutic approaches is urgently needed for elderly patients with MMI.

The infarct volume of MMI has been reported to exceed 200 mL within 24 h of stroke onset and to rapidly increase to 460 mL from 24 to 48 h after stroke onset due to secondary malignant brain edema (11). Theoretically, timely removal of sufficient necrotic tissue from the core area of massive infarct within 24–48 h of onset can effectively attenuate edema, increase compensatory intracranial space, and prevent the occurrence of cerebral hernia. We have reported that treatment of MMI using catheter-based aspiration of necrotic tissue can avoid severe trauma and other surgery-related risks caused by DHC and has achieved good results (12). However, there are limited controlled studies on the internal decompression technique alone without bone flap removal in the treatment of MMI (9, 12).

Therefore, this study aimed to investigate the efficacy and safety of stereotactic aspiration of necrotic brain tissue for the treatment of MMI in patients aged 61 years or older, by comparing it with the optimal medical conservative treatment.

## 2 Materials and methods

### 2.1 Patients

**Figure 1:**
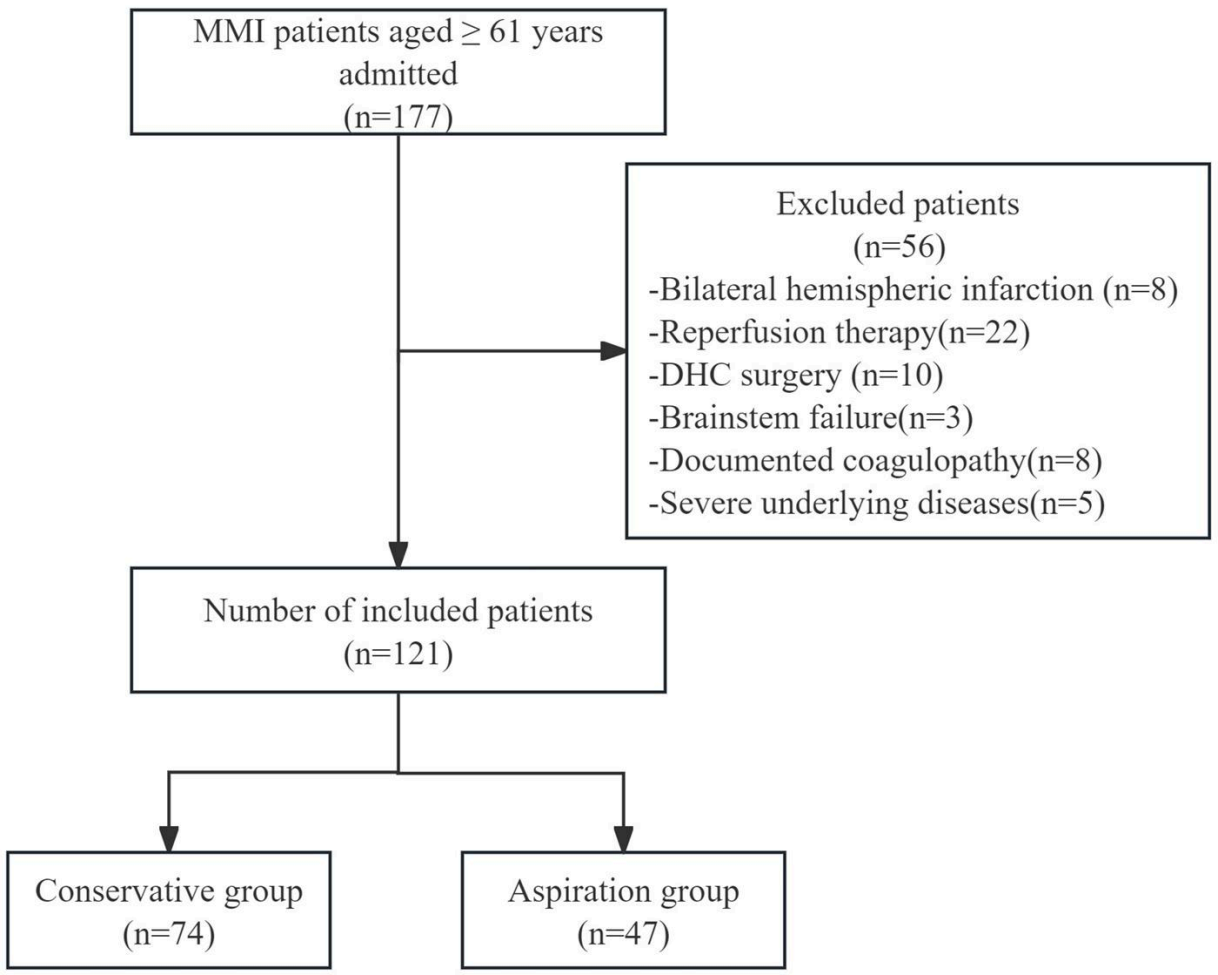
Study enrollment precess. MMI, malignant middle cerebral artery infarction; DHC, decompressive hemicraniectomy.

A retrospective cohort study was conducted. We enrolled a total of 121 consecutive patients with MMI aged 61 years or older. They were admitted to three medical centers-Yancheng Third People's Hospital, Yancheng No.1 People's Hospital and Huai'an First People's Hospital-from January 2016 to May 2024.The total number of screened patients and the screening procedures are depicted in Figure 1. This study was approved by the Ethics Committees of Yancheng Third People's Hospital (Ethics No.: Lun Shen-2022-30, approved on March 30, 2022), Yancheng No. 1 People's Hospital (Ethics No.: 2016-K003, approved on February 1, 2016), and Huai'an First People's Hospital (Ethics No.: JS-2022-001-01, approved on May 13, 2022). The eligible subjects were divided into two groups, namely, the conservative treatment group and the aspiration group according to different treatment methods. The decision to receive aspiration therapy was primarily based on the clinical judgment of the doctor and the assessment by the multidisciplinary team.

The inclusion and exclusion criteria in this study were formulated with reference to the protocols of DESTINY Ⅱ, et al. (5, 12, 13). Patients meeting the following criteria were eligible for inclusion: aged 61 years or older, with a pre-stroke score of 0 or 1 on the modified Rankin Scale (mRS), and presenting with an acute unilateral middle cerebral artery (MCA) infarction with a National Institutes of Health Stroke Scale (NIHSS) score above 14 (for infarcts of the nondominant hemisphere) or above 19 (for infarcts of the dominant hemisphere), either upon admission or after secondary deterioration. Additional radiological inclusion criteria were as follows: (1) proximal vessel occlusion [internal carotid artery (ICA) or M1 segment of the MCA] shown by CT angiography (CTA) or magnetic resonance angiography (MRA); and (2) massive MCA infarction encompassing at least two-thirds of the MCA territory confirmed by diffusion-weighted imaging (DWI) or computed tomography (CT) within 48 h of onset. In the aspiration group, the operation was performed between 24 and 72 h after stroke onset.

Patients were excluded if they presented with bilateral hemispheric infarction; received intravenous thrombolysis, mechanical thrombectomy, or DHC surgery; exhibited brainstem failure signs such as bilateral fixed and dilated pupils or a Glasgow Coma Scale (GCS) score ≤ 4; had documented coagulopathy or systemic bleeding disorders, severe underlying diseases impacting prognosis (e.g., advanced malignancy with expected survival <1 year, end-stage renal failure requiring dialysis, severe heart failure NYHA class IV, severe liver cirrhosis Child-Pugh class C, severe chronic obstructive pulmonary disease GOLD stage 4, or estimated life expectancy of less than 3 years) (5, 12, 13). For clinical safety concerns, all patients who underwent reperfusion therapy (intravenous thrombolysis or mechanical thrombectomy) were excluded from this study, regardless of treatment group assignment. This exclusion criterion applied to both the conservative treatment group and the aspiration group to avoid potential bias Both intravenous thrombolysis and mechanical thrombectomy can increase the risk of hemorrhage after successful recanalization. Aspiration therapy's primary safety concern is intraoperative bleeding, which currently lacks reliable management methods during the procedure. Therefore, for clinical safety, all patients who underwent reperfusion therapy were excluded from this study. The patients undergoing aspiration procedures were required to have preoperative CTA. If large vessel recanalization was detected, they were excluded from the study in order to minimize bleeding risk during the operation.

### 2.2 Surgical procedure of aspiration

**Figure 2:**
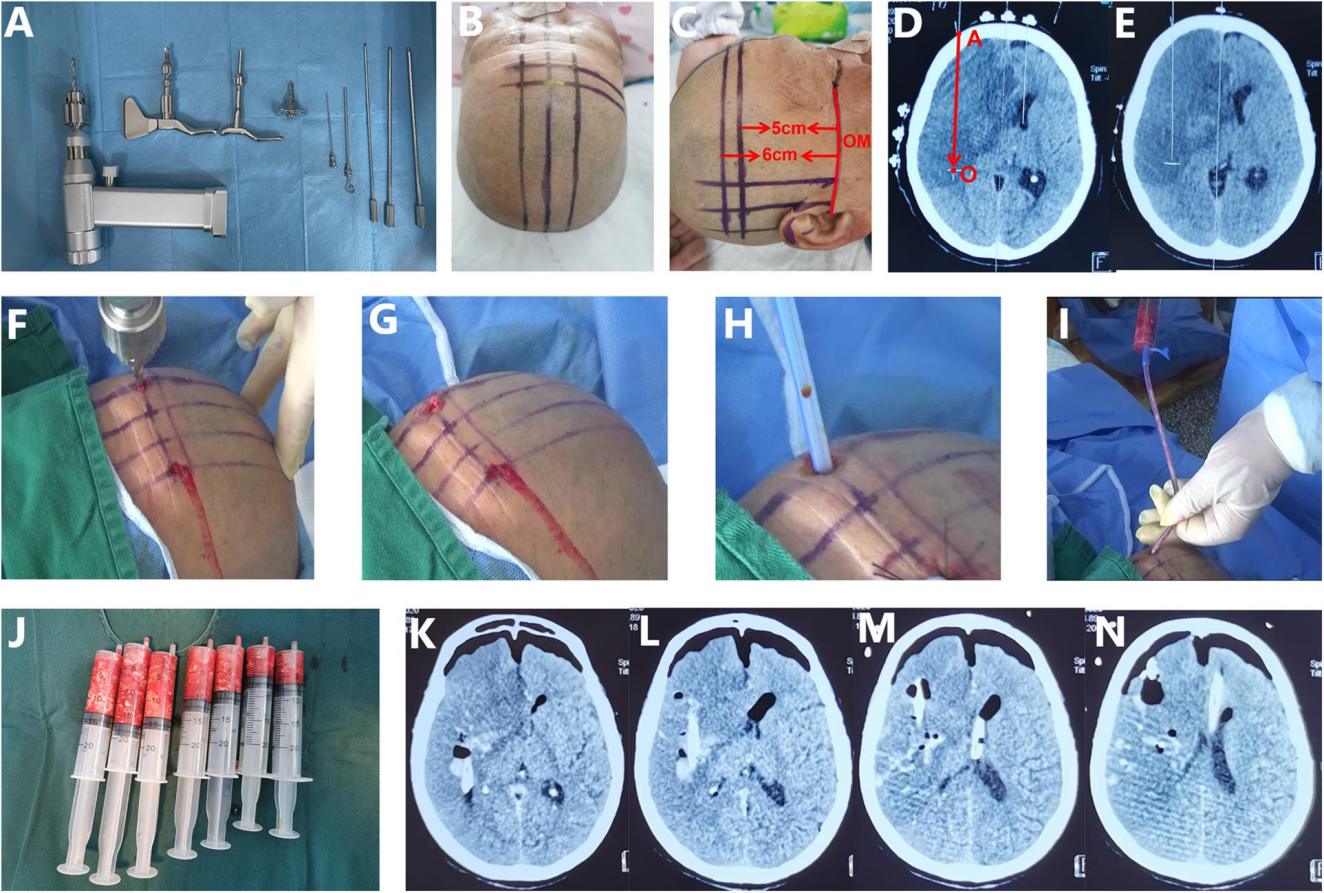
Principle and procedure of cubic stereotactic aspiration of necrotic brain tissue. **(A)** The main surgical instruments include an electric cranial drill. **(B,C)** The median sagittal line, the orbitomeatal (OM) line and the horizontal line 5 cm above the OM line were drawn. **(D,E)** A standard CT slice was obtained through several metal markers fixed on the horizontal line 5 cm above the OM line. The slice showing the largest infarct size was identified at approximately the same level. The puncture pathway “AO” in this case was located on the paramedian sagittal plane, 3.5 cm lateral to the median sagittal line and, on the horizontal plane 5 cm above the OM line. **(D–G)** A 6 mm bone hole was drilled with an electric cranial drill along a trajectory defined by two mutually perpendicular planes: the horizontal and the parasagittal planes. **(H)** A 12-French silicone catheter was inserted into the deep part of the infarct lesion. **(I)** Necrotic brain tissue was aspirated with a 20 mL syringe at a negative pressure of 5 mL from deep to shallow along the puncture pathway. **(J)** Approximately 70 mL of necrotic tissue was aspirated. **(K–N)** The immediate postoperative CT scan revealed bilateral collapsed prefrontal tissue, intracranial gas, and reduced midline shift, indicating adequate decompression by aspiration.

Once the subjects were selected for this study, they were given standard basic treatments. These included airway management and mechanical ventilation; osmotherapy using mannitol, glycerol, or diuretics; blood pressure control; and nutritional support. All treatments followed the guidelines developed by the Neurocritical Care Society (9) and the quality control standards of the Stroke Center of Jiangsu Province. Besides the above-mentioned standard basic treatments, all the patients in the aspiration group accross the three centers received cubic stereotactic aspiration developed by Sun (12, 14). The operative process was as follows (12, 14) (Figure 2).

First, we marked three reference lines on the scalp: the orbitomeatal (OM) line ipsilateral to the infarction, a horizontal line 5 cm above the OM line, and the midsagittal line (Figures 2B,C). Then, several reference metal electrodes were attached to the horizontal line 5 cm above the OM line. After that, a CTscan was perfomed again using the OM line as the baseline to obtain a standard CT image (Figure 2D). According to the re-scanned CT image, we identified the slice where the infarction was largest, approximately 5–6 cm above the OM line (Figures 2D,E). Based on the CT slice showing the largest infarct, we determined the puncture site on the surface of the skull, planed the puncture pathway, and measured the puncture depth range (Figure 2D).

Second, using the puncture parameters obtained from the CT image, we drew the puncture point on the scalp. Then we drew the intersection lines between the planes where the puncture pathway was located (horizontal plane, sagittal plane or coronal plane) and the scalp (Figure 2F). The puncture point was typically located on the forehead, 3.5 cm beside the median sagittal line. At this point, the puncture pathway was approximately located on the parasagittal plane 3.5 cm next to the median sagittal line and on the horizontal plane 5–6 cm above the OM line (Figure 2F). Based on the cubic stereotactic puncture principle developed by Sun (12, 14), accurate puncture of the target point was achieved by simultaneously puncturing along two mutually perpendicular planes, such as the horizontal plane and the sagittal plane in the case (Figure 2F). Notably, the planned puncture pathway should avoid passing through normal brain tissue (Figure 2D).

Third, under local anesthesia with lidocaine or general anesthesia, an electric cranial drill was used to make a bone hole with a diameter of 5–6 mm along the puncture direction (Figure 2G). Simultaneous puncture along two mutually perpendicular planes ensured accurate orientation (Figure 2F). Then, a 12- or 14-French silicone catheter was inserted into the deep infarct lesion. Necrotic brain tissue was aspirated from deep to shallow along the puncture pathway with a 20 mL syringe at a negative pressure of 5 mL (Figures 2H,I). The aspiration volume was controlled between 50 and 100 mL. After finishing the aspiration, normal saline with adrenaline was used as a rinse to prevent intraoperative bleeding until the rinse fluid became clear. Finally, the catheter was connected to a drainage bag to allow natural drainage of fluid. A CT scan should be performed within 6 h after the operation to evaluate the effect of aspiration and the extent of intraoperative bleeding.

Repeated aspiration could be conducted within five days after the procedure if CT scan showed evident mass effect and increased intracranial pressure (ICP). The decision to perform repeated aspiration was based on clinical symptoms (worsening neurological deficits, signs of increased ICP) and radiological findings (midline shift >5 mm, compressed ventricles). The frequency of repeated aspiration was typically once daily within 5 days post-procedure, depending on individual patient response. The drainage tube was typically removed within a period of 5 to 7 days following the procedure when the mass effect on the follow-up CT was relieved, and the intracranial pressure was controllable.

### 2.3 Study definitions and outcomes

The preoperative infarct volume was assessed 24–30 h after onset using CT or MRI-DWI. The volume was calculated employing the formula A × B × C/2, where A is the biggest diameter, B is the diameter perpendicular to A, and C is the number of slices multiplied by the slice thickness (15, 16). The definition of aspiration-related significant bleeding was established with reference to the standard of Parenchymal Hematoma type 2 (PH-2) bleeding in the ECASS II study (17). Significant hemorrhage in the infarct lesion during aspiration was defined as hemorrhage exceeding 15 mL or 20% of the volume of the suctioned tissue confirmed by CT. If the bleeding volume in the infarct area is less than 20% of the suctioned necrotic tissue, the net space-occupying effect, ICP and local compression of surrounding normal brain tissues are still reduced. Therefore, such bleeding may be considered clinically insignificant.

Short-term results included the incidence of cerebral hernia within 1 week and mortality, as well as the incidence of intracranial hematoma and intracranial infection within 30 days after stroke onset. All subjects were followed up for 6months by telephone or in the outpatient clinic. The long-term efficacy was evaluated utilizing mRS at 6 months. A favorable outcome was defined as mRS ranging from 0 to 3, and an unfavorable outcome was defined as mRS ranging from 4 to 6. Non-severely disabled survival was defined as mRS from 0 to 4 (2, 5, 13, 18, 19). The primary efficacy endpoint was the ratio of favorable outcome (mRS0-3). Secondary endpoints included the ration of survival without severe disability (mRS0-4), cumulative mortality and mRS distribution, etc.

### 2.4 Statistical analysis

The data were analyzed statistically using SPSS version 26.0 and R software version 4.3.0. Data were presented as *n* (%) or mean ± standard deviation (SD). Categorical data were analyzed using Pearson's chi-square test or Fisher's exact test for small sample. Continuous data were analyzed using the Student's *t* test. Ordinal data with multiple ranks were analyzed using the Mann–Whitney *U* test. A binary logistic regression model was used to analyze the effects of aspiration therapy on 6-month favorable outcomes (mRS 0–3). First, possible factors affecting favorable and unfavorable prognosis were analyzed by univariate analysis. The factors included gender, age, smoking history, drinking history, atrial fibrillation, hypertension, diabetes, GCS, NIHSS 24 h after onset, infarct volume 24–30 h after onset, and treatment methods (20). Then variables with *P* < 0.05 in univariate analysis and treatment methods were included in the binary logistic regression model to analyze the effects of the two different treatment methods on long-term prognosis. Ordinal logistic regression (proportional odds model) was further performed to evaluate the association between treatment methods and the entire ordinal mRS distribution (0–6). Proportional odds ratios (PORs) with 95% CIs were reported. Differences were considered significant at *P* < 0.05 (two-tailed).

## 3 Results

### 3.1 Baseline data between aspiration and conservative treatment groups

Baseline data for aspiration and conservative treatment groups are listed in Table 1. No significant differences were observed between the two groups in terms of age, gender (male), smoking history, drinking history, diabetes, atrial fibrillation, time from onset to hospital arrival, time from onset to CT scan, GCS and NIHSS scores at admission and 24 h after stroke onset, infarct site, midline shift, infarct volume at 24–30 h after stroke onset (*P* > 0.05). The mean time from stroke onset to operation was 34.54 ± 8.92 h. The proportion of patients with hypertension history was 52.7% in the conservative group and 27.7% in the aspiration group (*χ*^2^ = 7.356, *P* = 0.007).

**Table 1:** Comparison of the basic characteristics of the conservative treat group and the aspiration group.

Characteristics	Conservative group (*n* = 74)	Aspiration group (*n* = 47)	Statistics	*P* value
Age	72.28 ± 7.422	70.26 ± 6.863	*t* = 1.508	*P* = 0.134
Gender(male)	41 (55.4%)	27 (57.4%)	*χ*^2^ = 0.049	*P* = 0.825
Smoking history	30 (40.5%)	20 (42.6%)	*χ*^2^ = 0.048	*P* = 0.827
Drinking history	8 (10.8%)	9 (19.1%)	*χ*^2^ = 1.655	*P* = 0.198
Hypertension	39 (52.7%)	13 (27.7%)	*χ*^2^ = 7.356	*P* = 0.007
Diabetes	35 (47.3%)	21 (44.7%)	*χ*^2^ = 0.079	*P* = 0.778
Atrial fibrillation	40 (54.1%)	27 (57.4%)	*χ*^2^ = 0.134	*P* = 0.714
Time from onset to hospital arrival(h)	6.10 ± 2.11	6.51 ± 3.10	*t* = 0.849	*P* = 0.397
Time from onset to admission CT(h)	6.63 ± 2.14	6.96 ± 3.10	*t* = 0.707	*P* = 0.481
GCS at admission	10.65 ± 1.65	10.45 ± 1.77	*t* = 0.638	*P* = 0.525
NIHSS at admission	23.30 ± 5.65	24.51 ± 6.58	*t* = 1.080	*P* = 0.283
GCS at 24 h after stroke onset	9.51 ± 2.634	9.34 ± 3.081	*t* = 0.742	*P* = 0.742
NIHSS at 24 h after stroke onset	25.11 ± 5.641	26.64 ± 6.716	*t* = 0.180	*P* = 0.180
Infarct volume in 24–30 h after stroke onset (mL)	233.54 ± 53.565	245.19 ± 62.667	*t* = 0.277	*P* = 0.277
Midline shift in 24–30 h after stroke onset (mm)	12.19 ± 1.46	12.53 ± 1.33	*t* = 1.302	*P* = 0.195
The occurrence of cerebral hernia at 24 h after stroke onset	0 (0%)	2 (4.3%)	-	*P* = 0.149^a^
intracranial hematoma	0 (0%)	0 (0%)	-	-
Mean time from stroke onset to operation(h)	-	34.54 ± 8.921	-	-

^a^ Fisher's exact test.

### 3.2 Perioperative outcomes between aspiration and conservative treatment groups

In the aspiration group, initial aspirate volume ranged from 50–100 mL with a mean of 75.25 ± 15.87 mL. Postoperative midline shift measured 10.65 ± 1.36 mm, demonstrating a significant reduction compared to the preoperative measurement of 12.53 ± 1.33 mm (*t* = 32.38, *P* < 0.001). In the aspiration group, 4 patients underwent single aspirations, 43 required 2 to 4 aspirations. Table 2 lists the cumulative mortality rate, incidence of cerebral hernia, and major complications within 30 days after stroke onset for the two groups. The early incidence of cerebral hernia was 47.3% in the conservative group and 8.5% in the aspiration group (*χ*^2^ = 19.797, *P* < 0.001). The one-month mortality rates were 64.9% in the conservative group and 19.1% in the aspiration group (*χ*^2^ = 24.110, *P* < 0.001). There was no statistically significant difference between the two groups in the incidence of postoperative intracranial hematoma and infection (*P* *>* 0.05).

**Table 2:** Comparison of the major complications and mortality between the conservative treat group and the aspiration group within 30 days after the stroke onset.

Outcomes	Conservative group (*n* = 74)	Aspiration group (*n* = 47)	Statistics	*P* value
Early intracranial hematoma	3 (4.1%)	3 (6.4%)	*χ*^2^ = 0.331	*P* = 0.565
Early cerebral hernia	35 (47.3%)	4 (8.5%)	*χ*^2^ = 19.797	*P* < 0.001
Intracranial infection	0 (0%)	2 (4.3%)	-	*P* = 0.149^a^
Mortality	48 (64.9%)	9 (19.1%)	*χ*^2^ = 24.110	*P* < 0.001

^a^ Fisher's exact test.

### 3.3 Follow-up outcomes between aspiration and conservative treat groups

The follow-up outcomes at 6 months after stroke onset are shown in Table 3. The full distribution of mRS scores (0–6) for each group is presented, showing that aspiration therapy significantly improved mRS score distribution compared to conservative treatment (Z = −5.085, *P* < 0.001 by Mann–Whitney *U* test; POR = 6.737, 95% CI 3.18–14.27, *P* < 0.001 by ordinal logistic regression). The 6-month mortality in the aspiration group was significantly lower than that in the conservative treatment group (27.7% vs. 71.6%, *P* < 0.001). Additionally, the proportions of patients with mRS scores 0–4 (55.3% vs. 17.6%, *P* < 0.001) and 0–3 (25.5% vs. 2.7%, *P* < 0.001) were significantly higher in the aspiration group.

**Table 3:** Comparison of the scores on the mRS and the mortality between the conservative treat group and the aspiration group at 6 months after the stroke onset.

mRS	Conservative group (*n* = 74)	Aspiration group (*n* = 47)	Statistics	*P* value
0	0 (0%)	0 (0%)	*Z* = −5.085	*P* < 0.001
1	0 (0%)	0 (0%)		
2	0 (0%)	0 (0%)		
3	2 (2.7%)	12 (25.5%)		
4	11 (14.9%)	14 (29.8%)		
5	8 (10.8%)	8 (17.0%)		
6	53 (71.6%)	13 (27.7%)		
Mortality	53 (71.6%)	13 (27.7%)	*χ*^2^ = 22.406	*P* < 0.001
mRS 0–4	13 (17.6%)	26 (55.3%)	*χ*^2^ = 18.755	*P* < 0.001
mRS 0–3	2 (2.7%)	12 (25.5%)	*χ*^2^ = 14.641	*P* < 0.001

### 3.4 Univariate analysis of the 6-month unfavorable outcomes (mRS 4–6)

According to the mRS score at 6 months, the subjects were divided into two groups: the favorable outcome group (mRS 0–3) and the unfavorable outcome group (mRS 4–6). The favorable group included 14 patients, while the unfavorable group included 107 patients. Table 4 presents the univariable analysis of factors assocaiated with unfavorable outcome (mRS 4–6). The proportion of patients treated with medical therapy alone was significantly higher in the unfavorable outcome group compared to those treated with aspiration (67.3% vs. 32.7%, *P* < 0.001). The unfavorable outcome group had a higher prevalence of atrial fibrillation (59.8% vs. 21.4%, *P* = 0.007), smoking (44.9% vs. 14.3%, *P* = 0.029), diabetes (50.5% vs. 14.3%, *P* = 0.011). Additionally, they showed lower GCS scores (9.22 ± 2.710 vs. 11.14 ± 3.035, *P* = 0.015) and higher NIHSS scores (26.10 ± 5.972 vs. 22.64 ± 6.428, *P* = 0.046) at 24 h after onset compared to the favorable outcome group. No statistically significant differences were observed between the two groups in terms of age, sex, hypertension, or infarction volume at 24–30 h after onset (*P* > 0.05).

**Table 4:** Univariable analysis that affects unfavorable prognosis (mRS 4–6).

Variables	Favourable prognosis	Unfavorable prognosis	Statistics	*P* value
	(*n* = 14)	(*n* = 107)		
Age	70.29 ± 7.426	71.65 ± 7.246	*t* = −0.663	*P* = 0.509
Gender(male)	9 (64.3%)	59 (55.1%)	*χ*^2^ = 0.421	*P* = 0.517
Smoking history	2 (14.3%)	48 (44.9%)	*χ*^2^ = 4.773	*P* = 0.029
Drinking history	2 (14.3%)	15 (14.0%)	*χ*^2^ = 0.001	*P* = 0.978
Hypertension	7 (50.0%)	45 (42.1%)	*χ*^2^ = 0.319	*P* = 0.572
Diabetes	2 (14.3%)	54 (50.5%)	*χ*^2^ = 6.519	*P* *=* 0.011
Atrial fibrillation	3 (21.4%)	64 (59.8%)	*χ*^2^ = 7.381	*P* *=* 0.007
GCS at 24 h after stroke onset	11.14 ± 3.035	9.22 ± 2.710	*t* = 2.457	*P* = 0.015
NIHSS at 24 h after stroke onset	22.64 ± 6.428	26.10 ± 5.972	*t* = −2.021	*P* = 0.046
Infarct volume in 24–30 h after stroke onset (mL)	214.86 ± 48.165	241.10 ± 57.897	*t* = -1.623	*P* = 0.107
Midline shift in 24–30 h after stroke onset (mm)	11.64 ± 1.39	12.41 ± 1.40	*t* = 1.932	*P* = 0.056
Therapy method				
Aspiration group	12 (85.7%)	35 (32.7%)		
Conservative group	2 (14.3%)	72 (67.3%)	*χ*^2^ = 14.641	*P* < 0.001

### 3.5 Multivariate logistic regression analysis of outcomes

Variables with *P* < 0.05 in the univariate analysis of prognosis in Table 4, including smoking, diabetes, atrial fibrillation, GCS score and NIHSS score at 24 h after onset, as well as the treatment modality (conservative treatment or aspiration) were included in a binary logistic regression mode. This model analyzed the independent factors affecting the 6-month unfavorable outcome (mRS 4–6) (Table 5). The results demonstrated that smoking, diabetes, atrial fibrillation, and additionally treatment modality remained independent factors influencing unfavorable prognosis (mRS 4–6) in MMI patients (*P* < 0.05) (seen in Table 5).

**Table 5:** Binary and ordinal logistic regression analyses of factors associated with unfavorable prognosis and mRS distribution.

Variables	Binary logistic regression		Ordinal logistic regression	
	OR (95% CI)	*P* value	POR (95% CI)	*P* value
Smoking history	20.065 (1.681–239.475)	0.018	2.217 (1.008–4.872)	0.048
Diabetes	17.342 (1.850–162.560)	0.012	1.48 (0.683–3.211)	0.320
Atrial fibrillation	7.473 (1.249–44.699)	0.028	3.282 (1.418–7.596)	0.006
GCS at 24 h after stroke onset	0.804 (0.468–1.382)	0.430	0.698 (0.541–0.902)	0.006
NIHSS at 24 h after stroke onset	1.089 (0.823–1.440)	0.551	0.946 (0.845–1.059)	0.338
Therapy method				
Aspiration group (Reference)	-	-	-	-
Conservative group	51.713 (5.029–531.748)	<0.001	11.290 (4.827–26.409)	<0.001

OR, odds ratio; CI, confidence interval; POR, proportional odds ratio.

GCS, Glasgow Coma Scale; NIHSS, National Institutes of Health Stroke Scale.

To further validate the robustness of our primary finding, an ordinal logistic regression (proportional odds model) was performed to assess the effect of treatment method on the entire mRS distribution. In the univariable ordinal logistic regression model, the POR for the conservative group vs. the aspiration group was 6.737 (95% CI: 3.181–14.271, *P* < 0.001). After adjusting for smoking, diabetes, atrial fibrillation, GCS and NIHSS scores at 24 h after onset in the multivariable ordinal logistic regression model, the POR for the conservative group vs. the aspiration group was 11.290 (95% CI: 4.827–26.409, *P* < 0.001) (seen in Table 5). These findings were consistent with the primary analysis (Mann–Whitney *U* test for mRS distribution and binary logistic regression for dichotomous prognosis), collectively indicating that conservative treatment was significantly associated with worse mRS outcomes compared to aspiration therapy.

## 4 Discussion

In this study, we found that stereotactic aspiration of necrotic brain tissue significantly reduced the incidence of early cerebral hernia and mortality in elderly patients with MMI. It also improved the 6-month proportion of survival, favorable outcome (mRS 0–3) and survival without severe disability (mRS 0–4) without increasing the risk of bleeding compared with medical treatment alone. In addition, both binary and ordinal logistic regression analyses showed that aspiration was an independent influencing factor for better outcomes in elderly MMI patients. Compared with DESTINY-II study (5), aspiration therapy appears to be superior or equivalent to DHC in improving the 6-month rate of survival, favorable outcome (mRS 0–3), and survival without severe disability (mRS 0–4) for MMI patients older than 60 years. Taken together, these findings suggest that stereotactic aspiration of necrotic brain tissue can reduce mortality and improve long-term prognosis in the elderly patients with MMI.

**Figure 3:**
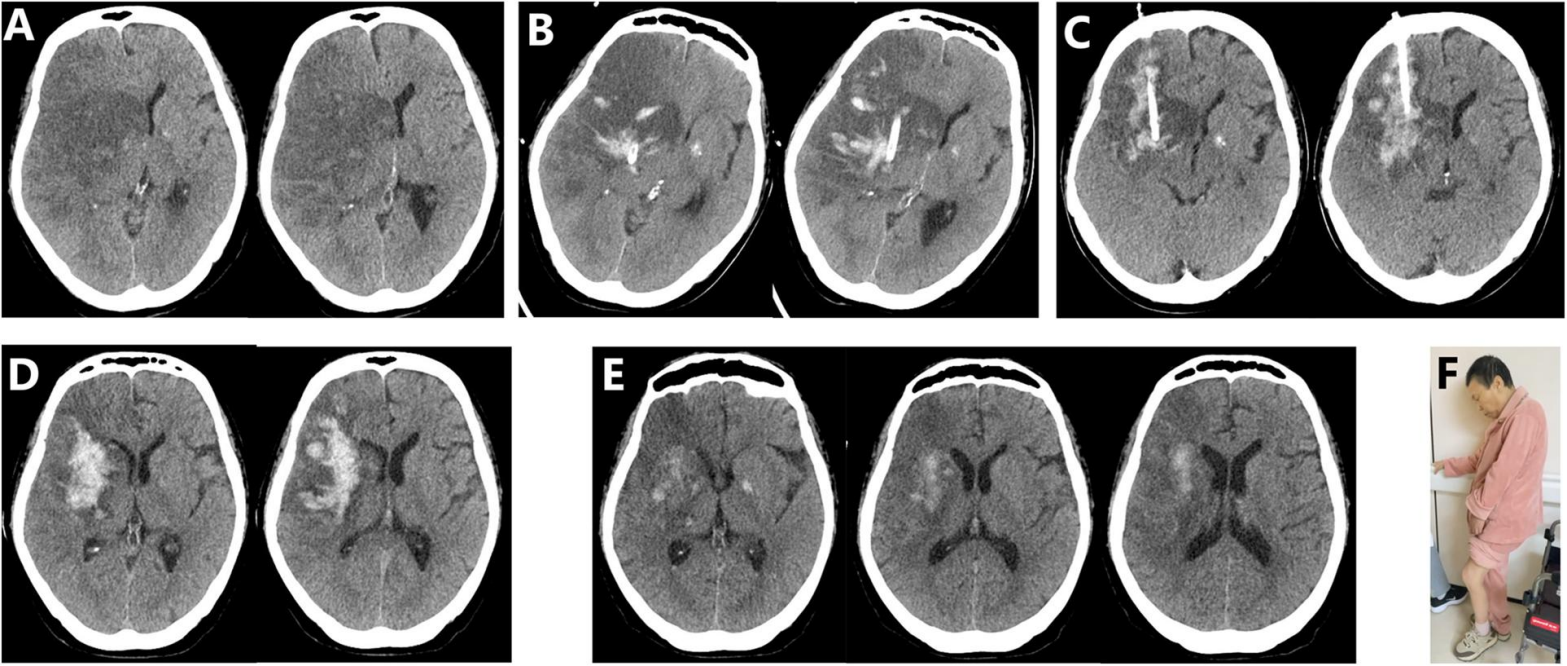
Surgical procedure and postoperative follow-up of a 70-year-old female patient with malignant middle cerebral artery infarction. **(A)** Preoperative computed tomography (CT) showed a 13 mm midline shift and a completely compressed lateral ventricle ipsilateral to the infarction. **(B)** CT scan on day 1 after the operation showed that the midline shift did not worsen, but the mass effect was obvious. Subsequently, necrotic brain tissue was aspirated again. **(C)** CT scan on day 3 after the operation showed that the mass effect was relieved. **(D)** CT scan on day 6 after the operation showed that the midline shift had almost returned to normal. There was a small amount of bleeding within the infarct area, but no mass effect was observed. **(E)** CT scan 2 weeks after the operation showed that the midline shift fully recovered and the hemorrhage in the infarct area was absorbed. **(F)** After 6 months of follow-up, the patient could walk independently using a cane and the mRS score was 3.

This study shows that the aspiration of necrotic brain tissue can significantly alleviate the mass effect, reduce ICP and prevent the formation of cerebral hernia. Some studies reported that standard DHC could create a compensatory volume of about 90 mL (21). However, the infarct volume in patients with MMI within 24 h after stroke onset generally exceeds 200–240 mL and reaches up to 461 mL due to explosive edema growth 24–48 h after onset, which provides strong rationale for treating MMI by aspiration (11). In this study, we found that an initial aspiration volume of 70–100 mL, accounting for about 1/3 of the total infarct volume, could effectively relieve peak edema and prevent cerebral hernia. Nevertheless, we found that aspirating such a large amount of early necrotic brain tissue was technically challenging. To aspirate necrotic brain tissue as much as possible, we performed aspiration from deep to shallow along the long axis of infarction. If the suction volume does not reach 70 mL, two bone holes may need to be drilled to place two catheters for aspirating more necrotic brain tissue. Repeated aspiration may be required within 5 days after the procedure if CT confirms recurrence of evident mass effect and ICP increases significantly. Figure 3 shows an example of stereotactic aspiration for treating MMI.

Intraoperative bleeding is the main concern of aspiration therapy. However, our study did not show that early aspiration increased the risk of significant bleeding. The rate of significant bleeding associated with aspiration was 6.8%, which was not significantly different from that of medical treatment alone in this study, but was lower than that of DHC (20.7%) (5). Several factors explain the low bleeding risk associated with aspiration. First, the unique pathophysiological mechanism of MMI, which differs from LHI without malignant edema plays a major role. Severe lack of collateral circulation in patients with MMI results in more extensive infarct areas with no blood flow; and this is the main reason that aspiration does not increase the risk of bleeding. Second, the timing of aspiration in this study avoided the period of hemorrhagic transformation of LHI (4–7 days after onset) and the time window of spontaneous vascular recanalization (3 days after onset) (22, 23). Third, all patients in this study underwent preoperative CTA to confirm occlusion of the feeding artery, thereby effectively controlling the risk of bleeding. Finally, surgical skills are also very important to prevent intraoperative bleeding. For example, the puncture point and puncture path were usually selected at the level of 5–8 cm above the OM line to avoid damage to the large vessels of the lateral fissure. The puncture pathway should avoid passing through normal brain tissue as much as possible, and the tip of the catheter must remain within the infarct area at all times during aspiration. Once fresh bleeding was observed during aspiration, the hematoma cavity was rinsed repeatedly with normal saline until the rinse fluid became clear and the drainage tube was left open for continuous drainage. If necessary, we injected urokinase into the hematoma cavity to liquify the hematoma and facilitate its clearance. In this study we found no life-threatening bleeding during the procedure.

**Figure 4:**
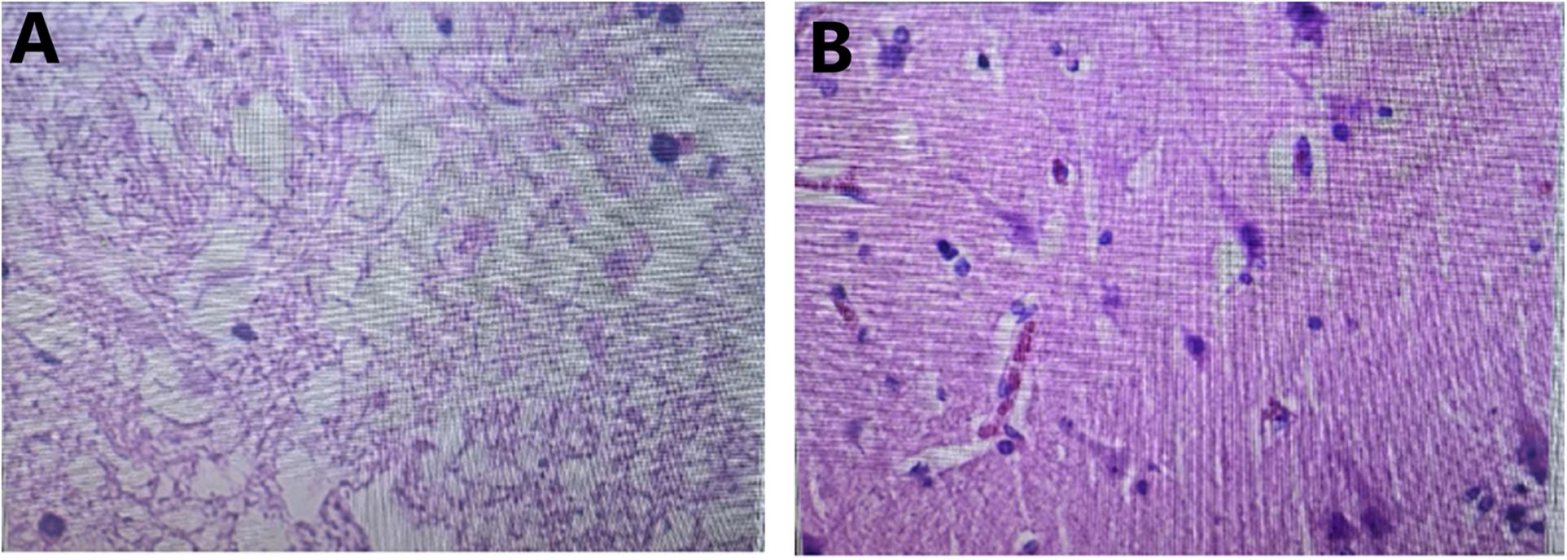
Pathological examination of cerebral tissue. **(A)** Necrotic cerebral tissue of a patient with malignant middle cerebral artery infarction by suction. **(B)** Normal cerebral tissue of a patient with cerebrovascular malformation.

Ethical concerns about aspiration of necrotic brain tissue need to be discussed. In particular, damage to viable tissue is an important ethical issue for aspiration. In patients with MMI, the infarct volume of 200–461 mL after 24 h of onset provides sufficient material basis for aspiration (11). The cubic stereotactic principle developed by Sun (12, 14) was used to accurately puncture the infarct lesions and avoid damage to the surrounding normal tissue. Based on the ischemic penumbra theory, it can be inferred that no viable neurons remains in the center of the infarct area 24 h after the onset of MMI (24, 25). We also confirmed this hypothesis by pathological examination of some aspirated specimens (Figure 4). On the other hand, for MMI patients over 60 years old, DHC treatment is not ideal. It has a mortality of up to 33%, and 80% of patients have an mRS Score of 5–6 at 6 months after stroke onset. Moreover, the treatment is extremely expensive (5). In this situation, less than 15% of patients with MMI undergo the procedure (9). Hence, it should be ethical for patients who refuse DHC to choose this simple and inexpensive aspiration technique without causing more damage than medical treatment alone.

However, this study has some limitations. First, a retrospective cohort study design was adopted. Thus, selection bias cannot be completely excluded. Fortunately, the baseline characteristics between the two groups in our study are almost balanced. This balance is possibly due to the strict inclusion and exclusion criteria and can reduce the bias. Second, due to the limited number of DHC cases in our centers and the exploratory nature of aspiration therapy for MMI, we chose conservative medical treatment as the control group instead of DHC. However, as DHC remains the standard treatment for elderly MMI patients, future comparative studies between aspiration therapy and DHC are still needed to confirm the safety and efficacy of aspiration therapy. Third, the sample size is small, and the duration of follow-up (six months) is short for MMI, a very serious disease. Fourth, the severe lack of blood flow and collateral circulation in MMI remains a theoretical hypothesis. In this study, CTA is used to evaluate the forward blood flow, but the data for the evaluation of collateral circulation are limited, which may increase the risk of intraoperative bleeding. Finally, the hypothesis that no viable neurons exist in the core infarct area of patients with MMI needs pathological comfirmation.

## 5 Conclusion

In conclusion, this study suggests that stereotactic aspiration of necrotic brain tissue is a safe and effective treatment for elderly patients with MMI. Importantly, stereotactic aspiration did not significantly increase the risk of clinically significant bleeding compared to conservative treatment. However, clinicians should remain vigilant about the potential for bleeding, particularly in patients with unexpected vessel recanalization. Future prospective, multicenter, randomized controlled trials comparing stereotactic aspiration with decompressive hemicraniectomy are warranted to establish the role of this minimally invasive technique in the treatment algorithm for elderly MMI patients.

## Data Availability

The original contributions presented in the study are included in the article/Supplementary Material, further inquiries can be directed to the corresponding authors.
